# Infection rate of *Eperythrozoon* spp. in Chinese population: a systematic review and meta-analysis since the first Chinese case reported in 1991

**DOI:** 10.1186/1471-2334-12-171

**Published:** 2012-07-31

**Authors:** De-Sheng Huang, Peng Guan, Wei Wu, Tie-Feng Shen, He-Ling Liu, Shuang Cao, Hao Zhou

**Affiliations:** 1 Department of Epidemiology, School of Public Health, China Medical University, Shenyang, 110001, China; 2 Department of Mathematics, College of Basic Medical Sciences, China Medical University, Shenyang, 110001, China; 3 Huludao Municipal Center for Disease Control and Prevention, Huludao, 125000, China; 4 Department of Biomedical Engineering, China Medical University, Shenyang, 110001, China

**Keywords:** *Eperythrozoon*, Infection, Meta-analysis

## Abstract

**Background:**

Eperythrozoonosis is an important animal health problem worldwide, it not only has a major impact on the economic viability, but also makes a significant impact on public health issues. The present systemic review intends to collate all relevant published data to assess the burden of *Eperythrozoon* infection in Chinese population and discuss the implications of these findings for public health policy.

**Methods:**

A meta-analysis was conducted to review the published studies that reported *Eperythrozoon spp.* in Chinese population. Inclusion criteria comprised of the use of microscopic venous blood smear examination for *Eperythrozoon* detection and a detailed description of sampling techniques.

**Results:**

Twenty-four cross-sectional studies with 52,433 participants and 14,951 positive cases, within the range of China mainland, were included in the present analysis. The infection rate of *Eperythrozoon* varied from 0 to 97.29% with geographical and seasonal variations, people with mild infection intensity contributed the major part (68.93%). The infection rates were highest in the children and adolescents group, significantly increased risk of *Eperythrozoon* infection was found among herdsmen.

**Conclusions:**

The current study raises awareness about the human eperythrozoonosis in China, which is a newly emerging zoonosis. The majority of *Eperythrozoon* infection intensity was asymptomatic mild infection. The infection rate of *Eperythrozoon* in Chinese population varied by geographical region, season, age and occupation. These factors need to be considered when conducting health education campaigns and comparing the surveillance results from different studies.

## Background

Eperythrozoonosis is an important animal health problem worldwide, more than 30 countries and regions have reported the diseases in at least 14 kinds of host animals in different species of vertebrate, including rodents, ruminants, and pigs [1-5]. It not only has a major impact on the economic viability (e.g. production losses, prevention or treatment costs and etc.), but also makes a significant impact on public health issues due to they have been judged to be a zoonosis and also an infectious disease transmissible from animals to humans [6,7].

The first recognized and confirmed human case of eperythrozoonosis was reported in 1986 worldwide [8]. The disease may manifest with fever, hemolytic anemia, swollen lymph nodes of the neck, an enlarged liver and spleen, leucopenia, neutropenia, thrombocytopenia and sometimes acute hemolysis, mild hepatitis and subclinical myocarditis [8-10]. Eperythrozoonosis actually is a microscopic diagnosis, and the causative agent was previously reported as ‘*Eperythrozoon*’. Currently, eperythrozoonosis was replaced by the laboratory diagnosis of haemotrophic mycoplasma infection. Haemotrophic mycoplasmas are small, pleomorphic, uncultivable bacteria which parasitise the surface of red blood cells of a wide range of mammalians and can induce erythrocytic deformity and damage [4,11]. Haemotrophic mycoplasmas were originally classified within two genera of the *Rickettsiales* order, i.e. *Eperythrozoon* and *Haemobartonella*. Then, based on strong phylogenetic evidence and 16 S ribosomal RNA gene sequences, *Eperythrozoon* and *Haemobartonella* were reclassified into the group of haemotrophic mycoplasmas within the family of *Mycoplasmataceae*[11-13].

However, the name of ‘*Eperythrozoon*’ and ‘Eperythrozoonosis’ is still adopted in the present study due to its wide acceptance and the cytology-based diagnosis in China. In China, the first human case was reported in 1991 [14], afterwards approximately 180 human cases have been sporadically reported in 18 provinces, autonomous regions and municipalities. Alongside animal field studies, several surveys have been conducted among Chinese populations to provide epidemiological knowledge of the distribution of *eperythrozoon* infection with the aim to lay the basis for disease prevention and control strategies [15-18]. Whilst those studies varied in infection diagnosis criteria or other methodological factors and were performed under diverse settings. Thus, different conclusions have been obtained, for example, some reported an increased risk in male, and others failed to confirm this association.

The present systemic review and meta-analysis takes advantages of the recent enrichment in the number of published investigations in China and intends to collate all relevant published data to assess the burden of *Eperythrozoon* infection in Chinese population and discuss the implications of these findings for public health policy.

## Methods

### Identification and eligibity of relevant studies

The literature was systematically reviewed by searching the ISI Web of Knowledge database, PubMed and the database of China National Knowledge Infrastructure (CNKI) for relevant articles without language restriction or publication year with the keywords “Eperythrozoon” (up to September 2011). The references cited in the retrieved publications were also screened to trace further relevant studies. Inclusion criteria comprised of the use of microscopic venous blood smear examination for *Eperythrozoon* detection (at least one *Eperythrozoon* per 20 vision fields of the microscope or per 200 erythrocytes), the inclusion of at least 75 people tested and a detailed description of sampling techniques. When studies from the same research group with overlapped population were found, only the one with larger population was included to avoid data duplication.

### Data extraction

Data were independently evaluated and extracted by two investigators (DSH and PG) with all the discrepancies discussed and resolved by consensus. For each included study, information was retrieved regarding publication characteristics (first author, journal name and year of publication); characteristics of participants (age, gender, occupation, number of people tested for *Eperythrozoon* infection, and number of positive cases), study characteristics (study sample type, period, sample collection method, detection method and criteria of positive diagnosis). The study sample type was classified into two categories, population-based or convenient (mainly including inpatients or outpatients) sample. The data quality of included studies was assessed and statistics were calculated again if applicable, the error corrections were made after enquiries from the authors or group discussion.

### Statistical analyses

The heterogeneity between the studies was evaluated by the Chi square-test based Q-statistic. The crude infection rate was calculated by pooling the number of *Eperythrozoon*-positive people by the total number of people tested from included studies. All the pooled statistics were calculated using data from population-based surveys. The infection rates were grouped into 3 levels by hierarchical cluster analysis. For age-specific analysis, the infection rate was compared within 4 broad age groups (≤19, 20–39, 40–59 and ≥60 years). The seasonal variations of the infection rate were examined within 2 major intervals (Winter & Spring: December-May; Summer & Autumn: June-November). Mantel-Haenszel chi-square test was adopted to analyze binomial data and logistic regression was performed for data with three or more categories, then the combined odds ratio was calculated. Due to the geographical variations, the combined odds ratio was adjusted for the area in the age and occupation-specific sub-analysis. The infection intensity was recorded according to Gulland’s method for animals [19], less than 30 *Eperythrozoon* infected in every 100 erythrocytes was classified as mild, between 30 and 60 termed as moderate and more than 60 recorded as the severe infection. Begg's test is used for the detection of publication bias. All analyses were done using SPSS software (SPSS 12.0 for windows, SPSS Inc., Chicago, IL, USA). All the *P* values were two-sided.

## Results

**Figure 1:**
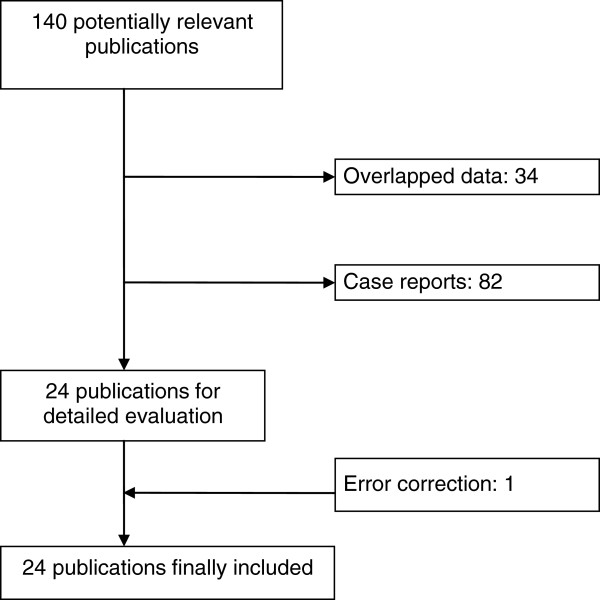
Identification of relevant publications, reasons for exclusion.

In total, 140 papers were evaluated from which 24 publications [15-36] were included (see Figure 1 for selection process). Table 1 shows crude *Eperythrozoon* infection rate in 52,433 people tested, with 14,951 positive individuals. The largest sample consisted of 18,316 tested people in Guangdong Province, followed by the study in Hubei Province (5,224 tested people) and in Shandong Province (5,217 tested people) and the study conducted by Chinese National Consortium on Eperythrozoonosis Research (CNCER) ranked 4^th^ from this sample size point of view (4,033 tested people). 

**Table 1:** **Infection rate of human* Eperythrozoon *in different areas of China**

	**First author, publication year**	**Province (city or county)**	**Sample type**	**Number of tested (N)**	**Number of positive (n)**	**Infection rate (%)**
1-3	Chinese National Consortium on Eperythrozoonosis Research (CNCER), 1995,1996 and 1997 [15-17]	Jiangsu	Population-based	1975	1552	78.58
		Hebei	Population-based	975	0	0.00
		Liaoning	Population-based	124	0	0.00
		Ningxia	Population-based	96	16	16.67
		Guangxi	Population-based	444	232	52.25
		Guangdong	Population-based	219	113	51.60
		Xinjiang	Population-based	200	90	45.00
4	Liu, 1997 [20]	Gansu	Population-based	277	205	74.01
			Convenient	737	649	88.06
5	Tai, 1998 [21]	Inner Mongolia	Population-based	1529	540	35.32
6	Huang, 1999 [22]	Yunnan (Yuxi)	Population-based	1461	984	67.35
			Convenient	3191	2486	77.91
7	Dong, 2000 [23]	Fujian	Population-based	932	434	46.57
			Convenient	54	51	94.44
8	Yang, 2000 [24]	Jiangsu	Population-based	210	42	20.00
			Convenient	190	13	6.84
9	Li, 2001 [25]	Anhui	Population-based	614	174	28.34
			Convenient	206	98	47.57
10	Zhao, 2001 [26]	Inner Mongolia	Convenient	187	153	81.82
11	Tao, 2001 [27]	Shandong	Population-based	776	57	7.35
12	Zhang, 2002 [28]	Ningxia	Population-based	150	56	37.33
			Convenient	150	67	44.67
13	Chen, 2003 [29]	Liaoning (Dalian)	Population-based	887	863	97.29
			Convenient	164	120	73.17
14	Zhou, 2003 [30]	Chongqing (Xingshan)	Population-based	174	17	9.77
			Convenient	407	2	0.49
15	Shi, 2007 [31]	Tibet	Population-based	3214	103	3.20
16	Zhou, 2007 [32]	Hubei (Xingshan county)	Population-based	5224	2931	56.11
17	He, 2007 [33]	Yunnan (Gongshan)	Population-based	1408	960	68.18
18	Li, 2008 [34]	Shandong (Wendeng)	Population-based	5217	15	0.29
19	Zhu, 2008 [35]	Shanghai	Population-based	997	129	12.94
20	Han, 2009 [36]	Shandong (Taian)	Population-based	617	85	13.78
			Convenient*	201	64	31.84
			Convenient	169	74	43.79
21	Qiu, 2010 [37]	Guangdong (Maoming)	Population-based	18316	1484	8.10
22	Huang, 2010 [38]	Guangxi (Mengshan)	Convenient	86	15	17.44
23	Deng, 2010 [39]	Zhejiang (Hangzhou)	Population-based	469	31	6.61
			Convenient*	111	23	20.72
24	Zhang, 2010 [40]	Liaoning (Huludao)	Population-based	75	23	30.67
		Total		52433	14951	28.51

*Here limited to the butcher, herdsman and veterinarian.

**Figure 2:**
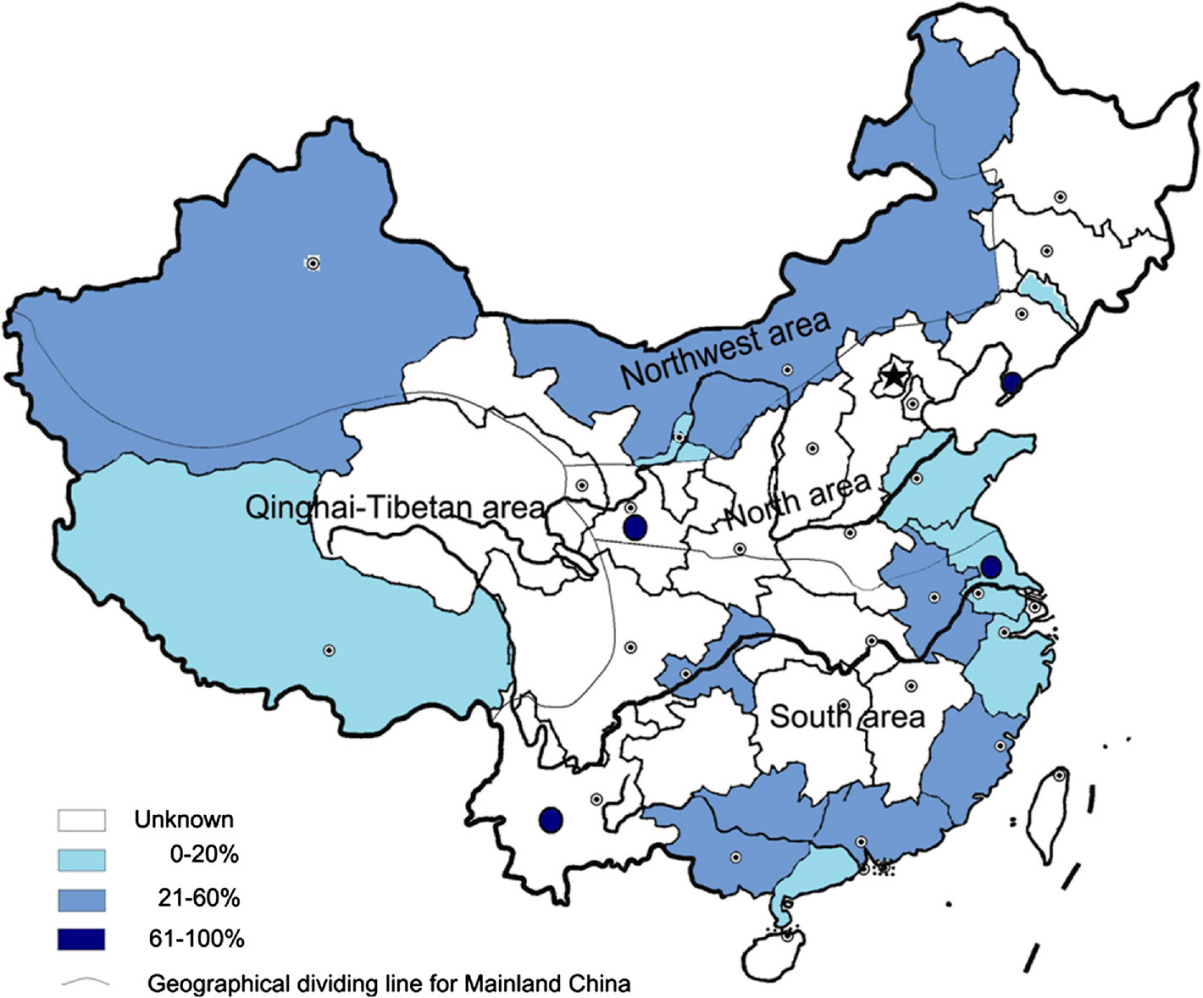
**Crude* Eperythrozoon *infection rate reported by the included population-based studies in mainland China**^**#**^**.** # The boundaries used in this map do not imply the expression of any opinion concerning the legal status of any territory, city or area of its authority or concerning the delimitation of its frontiers and boundaries.

Figure 2 shows the available crude *Eperythrozoon* infection rate among tested populations in different regions of China (using data from population-based surveys), by trisections of infection rate. The highest infection rate was found in farming and pastoral regions. Figure 2 also indicates the geographical heterogeneity of the infection rates, because there were also differences in some part of these regions.

Table 2 shows that there was no difference in the infection rate between the male and female (41.14% vs. 42.21%). Table 2 indicates the *Eperythrozoon* infection rates for 5 studies with age-specific data. The infection rates were highest in the children and adolescents group (younger than 19 years) and the infection rate decreased in 20–39, 40–59 year-groups, and the older age-groups (more than 60 years). From the 8 studies that provided occupation-specific infection information, there were remarkable differences in different occupations. The highest infection rate with statistical significance was found in herdsmen (Table 2). From the subset of studies with the information about contact history of animals, the infection rate was significant higher in the exposed group than that in the unexposed group (OR, 6.40 with the 95%CI 5.50-7.37). In the stratified analysis by nationality, there were only two studies with detail information about the nationality of the tested population, and no statistical differences were found for the * Eperythrozoon * infection rates.

**Table 2:** **Overall* Eperythrozoon *infection rate by selected variables**

**Variable**		**Number of countributing studies**	**Total tested**	***Eperythrozoon* positive**	**Crude infection rate (95% CI)**	**OR/Adjusted OR by area (95% CI)**	**Heterogeneity chi-square**	***P*value**	**Publication bias**
Gender	Male	8	7129	2933	41.14	Ref.	—	—	None
					(40.00 - 42.28)				
	Female	8	7408	3127	42.21	1.03	26.02	<0.01	
					(41.09 - 43.33)	(0.86 - 1.24)			
Age of enrolled population (yrs)	≤19	4	1771	1342	75.78	Ref.	—	—	†
					(73.78 -77.77)				
	20-39	5	9288	1738	18.71	0.55	67.52^#^	<0.01	
					(17.92 -19.51)	(0.48 - 0.64)			
	40-59	5	9330	1310	14.04	0.59	47.20 ^#^	<0.01	
					(13.34 -14.75)	(0.50 - 0.68)			
	≥60	5	3459	411	11.88	0.80	5.80^#^	0.02	
					(10.80 -12.96)	(0.67 - 0.96)			
	Age						94.47^#^	<0.01	
	Area						3980.23^#^	<0.01	
Occupation	Employees with the food industry exceptional	2	413	212	51.33	Ref.	—	—	†
					(46.51 - 56.15)				
	Employees in the food industry	3	1458	832	57.06	0.85	1.45^#^	0.23	
					(54.52 - 59.60)	(0.66 - 1.10)			
	Farmers	4	1551	754	48.61	1.98	4.78^#^	0.03	
					(46.12 - 51.10)	(1.07 - 3.64)			
	Herdsmen	6	530	296	55.85	3.65	27.34^#^	<0.01	
					(51.62 - 60.08)	(2.25 - 5.93)			
	Occupation						44.75^#^	<0.01	
	Area						641.56^#^	<0.01	
Contact history of animals	No	3	5548	575	10.36	Ref.	—	—	None
					(9.56 - 11.17)				
	Yes	3	1413	808	57.18	6.40	233.38	<0.01	
					(54.60 - 59.76)	(5.50 - 7.37)			
Season	Winter & Spring	2	9127	460	5.04	Ref.			†
					(4.59 - 5.49)				
	Summer & Autumn	2	10178	1564	15.37	4.26	6.91	<0.01	
					(14.67 - 16.07)	(3.01 - 6.02)			
Location	Urban area	2	839	633	75.45	Ref.	—	—	†
					(72.54 - 78.36)				
	Rural area	2	1456	1190	81.73 (79.75 - 83.71)	2.83 (0.36 - 22.55)	21.86	<0.01	

# Wald Chi-square; † Unable to evaluate.

The seasonal variations of the *Eperythrozoon* infection rates were evaluated in 2 included studies. The infection rate was significantly higher in summer & autumn than that in winter & spring (Table 2). Two studies provided the infection information separated into urban and rural areas, residents in the rural area had higher infection rates compared with residents in urban area, however, the difference was not statistically significant (OR, 2.83 with the 95%CI 0.36-22.55).

With respect to the route of transmission, there were three studies with interest on the route of vertical transmission, among the total 167 *Eperythrozoon*-positive mothers, 165 children tested positive for *Eperythrozoon*.

Table 3 shows the distribution of the *Eperythrozoon* infection intensity, among the 6,180 *Eperythrozoon*-positive individuals from 8 studies, people with mild infection intensity contributed the major part (68.93%), followed by moderate (19.21%) and severe infection (11.86%).

**Table 3:** **Distribution of* Eperythrozoon *infection intensity**

**Study**		**Number of positive**	**Mild**	**Moderate**	**Severe**
Tai, 1998 [21]		540	484	43	13
Zhao, 2001 [26]		153	98	39	16
Tao, 2001 [27]		57	44	11	2
Chen, 2003 [29]		866	367	157	342
Zhou, 2007 [32]	Male	1295	963	240	92
	Female	1636	1229	304	103
Zhu, 2008 [35]		129	129	0	0
Qiu, 2010 [37]	Symptomatic group	178	66	73	39
	Asymptomatic group	1301	855	320	126
Deng, 2010 [39]		25	25	0	0
Total (proportion)		6180 (100%)	4260 (68.93%)	1187 (19.21%)	733 (11.86%)

## Discussion

In past 20 years, there is increasing concern in human eperythrozoonosis, which is a newly emerging disease in China. Among the 140 publications that we have collected, eighty-two publications were the case reports of human eperythrozoonosis and nine of their titles indicated ‘the first case’ in their local area. Eperythrozoonosis has a wide spectrum of clinical manifestations, which can vary from subclinical infection to weakness, fever, icterus, anemia, and et al. For the prevention and control of this kind of disease, the infection distribution, related risk factors and possible routes of transmission are necessary. Thus, the published cross-sectional surveys of *Eperythrozoon* infection rates were summarized to provide a rough estimation of the above information. To our knowledge, this is the first systematic review about the epidemiological data on the infection rate of *Eperythrozoon* in China nationwide.

Within the range of China mainland, human eperythrozoonosis cases from 18 provinces, autonomous regions and municipalities have been reported, the infection rates varied from 0 to 97.29% in the 24 included studies, which indicated that several aspects of factors might contribute to the variations. From the geographical point of view, the relatively high infection rate was found in pastoral areas located in the northweast region, the lowest was found in Tibet and the major part of infection was asymptomatic mild infection. The great difference contributed our decision to compare the infection rates stratified by the selected factors after adjusting the geographical location.

It has been reducted from animal experiments that the transmission of eperythrozoonosis could be via the respiratory and gastrointestinal tracts and the vectors such as the mosquitoes were also involved in the eperythrozoonosis transmission [41]. This correspondence is also present in our results, the eperythrozoonosis existed all over the year while with seasonal distribution, which might due to seasonal exposure possibilities and densities of insect vectors.

As for the association between population characteristics and *Eperythrozoon* infection rate, our results are in partial concordance with the results from Chinese National Consortium on Eperythrozoonosis Research published in 1997 [17]. They indicated that the state of human body had the effect on the *Eperythrozoon* infection and no association between the gender and the infection rate was observed. The national survey showed that the infection rate was highest among the children and adolescents group and among the milking workers and doctors, nevertheless, the differences were not statistically significant, which is different from our results. This may due to that more people were analyzed in the present review and the selection bias might be reduced by including the people from broader geographical range. In the age-specific subanalysis, the highest infection rate was found in the children group, and in some studies, the second peak in older people was also observed, the pattern may attribute to their unmature or weak immune function. The results may also help the clinicians or pediatricians to keep the *Eperythrozoon* infection in mind, especially in the high-risk area. The occupational variations of infection rates could be attributed to the different exposure possibilities and extent. Those variations of *Eperythrozoon* infection rates in geographical location, age-group and occupation can have significant implications for the design and effectiveness of prevention and control strategies.

Among the collected publications, familial aggregation was reported by 3 individual studies, which suggested that the environmental factors or life style factors might be involved in the *Eperythrozoon* infection. Contact with livestock, or poultry and international travel have been reported as risk factors among those case report publications [9,10,42]. According to the available *Eperythrozoon* infection information in pigs or diary cows, multiple infection of different pathogens existed in eperythrozoonosis, which posed more threats for the farmers, herdsmen or milking workers. It has been summarized that Mycoplasma *suis* (formerly known as *Eperythrozoon suis*) can cause acute disease, but the major significance of M. *suis* infections lies in the fact that M. *suis* can establish chronic and persistent infections leading to a higher susceptibility to other infections, especially of the respiratory and digestive tracts [43].

Our study has several limitations. The major limitation is that in the included studies, the diagnostic method used to define *Eperythrozoon* infection is based on microscopy. The microscopic detection of the agent in blood smears is limited by its low sensitivity and specificity. [11,44,45]. It is difficult to differentiate *Eperythrozoon* from *haemobartonella*[11,46], there are reports on human haemotrophic mycoplasmas that seem to be bacteria which would be formerly classified as haemobartonella [9], thus all the reviewed articles might have suffered from misclassification bias. The second limitation or difficulty in the present systematic review is to deal with the heterogeneity of the included studies, in which the definition of *Eperythrozoon* infection, the sampling method and the representative of the population varied slightly from one to another. To accommodate the heterogeneity, only those studies using blood smear-based method and with detailed description of sampling techniques were included. The geographical distribution of the included tested population differed from the real-world distribution of population, thus it is difficult to derive the whole estimates of the *Eperythrozoon* infection rate in China by accounting for variation in study design and detection assays used. Third, we looked at a limited number of available variables and could not look at some factors such as socioeconomic factors and heath behaviours.

## Conclusions

In summary, the current study raises awareness about the human eperythrozoonosis in China, which is a newly emerging zoonosis. The majority of *Eperythrozoon* infection intensity was asymptomatic mild infection. The infection rate of *Eperythrozoon* in Chinese population varied by geographical region, season, age and occupation. These factors need to be considered when conducting health education campaigns and comparing the surveillance results from different studies.

## Competing interests

The authors declare that they have no competing interests.

## Authors’ contributions

DSH provided overall leadership to the study, secured funding and conducted the data collection and analysis. PG contributed to the data collection, data analysis and drafted the manuscript. WW, TFS, HLL, SC and HZ have been involved in the data collection, data check, data analysis and discussion. All authors contributed to the study design and participated in writing the paper. All authors have read and approved the final manuscript.

## Pre-publication history

The pre-publication history for this paper can be accessed here:

http://www.biomedcentral.com/1471-2334/12/171/prepub

## References

[B1] Berkenkamp SD Wescott RB Arthropod transmission of Eperythrozoon coccoides in mice Lab Animal Sci 1988 38 398 401 3184845

[B2] Hoff B Hood D McCaig L Moore A Eperythrozoonosis in sheep Can Vet J 1996 37 747 748 9111697 PMC1576680

[B3] Guimaraes AM Biondo AW Lara AC Messick JB Exploratory study of Mycoplasma suis (Eperythrozoon suis) on four commercial pig farms in southern Brazil Vet Rec 2007 160 50 53 17220522 10.1136/vr.160.2.50

[B4] Messick JB Hemotrophic mycoplasmas (hemoplasmas): a review and new insights into pathogenic potential Vet Clin Pathol 2004 33 2 13 15048620 10.1111/j.1939-165x.2004.tb00342.x

[B5] Wu J Yu J Song C Sun S Wang Z Porcine eperythrozoonosis in China Ann N Y Acad Sci 2006 1081 280 285 17135527 10.1196/annals.1373.038

[B6] Nikol'skiĭ SN Slipchenko SN Experiments in the transmission of Eperythrozoon ovis by the ticks H. plumbeum and Rh. bursa Veterinariia 1969 5 46 in Russian 5393434

[B7] Braverman Y Nematocera (Ceratopogonidae, Psychodidae, Simuliidae and Culicidae) and control methods Revue Scienti Techn 1994 13 1175 1199 10.20506/rst.13.4.819 7711309

[B8] Puntaric V Borcić D Vukelić D Jeren T Burek V Wikerhauser T Richter B Eperythrozoonosis in man Lancet 1986 2 868 869 2876318 10.1016/s0140-6736(86)92910-7

[B9] Steer JA Tasker S Barker EN Jensen J Mitchell J Stocki T Chalker VJ Hamon M A novel hemotropic Mycoplasma (hemoplasma) in a patient with hemolytic anemia and pyrexia Clin Infect Dis 2011 53 e147 151 22021921 10.1093/cid/cir666 PMC3205199

[B10] Bosnic D Baresic M Anic B Sentic M Cerovec M Mayer M Cikes N Rare zoonosis (hemotrophic mycoplasma infection) in a newly diagnosed systemic lupus erythematosus patient followed by a Nocardia asteroides pneumonia Braz J Infect Dis 2010 14 92 95 20428663 10.1590/s1413-86702010000100019

[B11] Hoelzle LE Haemotrophic mycoplasmas: recent advances in Mycoplasma suis Vet Microbiol 2008 130 215 226 18358641 10.1016/j.vetmic.2007.12.023

[B12] Messick JB Berent LM Cooper SK Messick JB Berent LM Cooper SK Development and evaluation of a PCR-based assay for detection of Haemobartonella felis in cats and differentiation of H. felis from related bacteria by restriction fragment length polymorphism analysis J Clin Microbiol 1998 36 462 466 9466759 10.1128/jcm.36.2.462-466.1998 PMC104560

[B13] Neimark H Johansson KE Rikihisa Y Tully JG Proposal to transfer some members of the genera Haemobartonella and Eperythrozoon to the genus Mycoplasma with descriptions of ‘Candidatus Mycoplasma haemofelis’, ‘Candidatus Mycoplasma haemomuris’, ‘Candidatus Mycoplasma haemosuis’ and ‘Candidatus Mycoplasma wenyonii’ Int J Syst Evol Microbiol 2001 51 891 899 11411711 10.1099/00207713-51-3-891

[B14] Tai XZ Yang DX Human eperythrozoonosis Chin Inner Mong Med J 1991 11 122 in Chinese

[B15] Shang DQ Li LY Luan JH Wang SY Zhang JZ Liu FC Zhang RY Li CY Cao L Zhao ZL Tang WY Jiang AG Wang YH Li GX Zhang FS Wang KJ Ma XH Jia ZL Zhang YQ Pei B Xu YC An epidemiological investigation of eperythrozoon infection in human and animals. A Collaborative Research Group on Eperythrozoonosis Zhonghua Liu Xing Bing Xue Za Zhi 1995 16 143 146 in Chinese 7648637

[B16] Shang DQ Li LY Pei B Wang SY Liu RH Zhao HY Sun GJ Tang HZ Tang AW Zhang JZ Sun ET Liu ZC Liu G Yang DD Liu FC An HZ Liang Q Li JG An epidemiological investigation of eperythrozoon infection in human and animals (II) Zhonghua Liu Xing Bing Xue Za Zhi 1996 17 221 224 in Chinese 9387587

[B17] Shang DQ Li YL Lu ZG Pei B Yan JH Li MR Xue ZQ Lin CM Gao J Zhang LH Xin SK Liu YE Liu YZ Gao WH Tao MK Yan YQ Cheng HF Chai SZ Yang H Zhang JC Wang DF Qi J An epidemiological investigation of eperythrozoon infection in human and animals (III) Zhonghua Liu Xing Bing Xue Za Zhi 1997 18 150 152 in Chinese 9812462

[B18] Yang D Tai X Qiu Y Yun S Prevalence of Eperythrozoon spp. infection and congenital eperythrozoonosis in humans in Inner Mongolia, China Epidemiol Infect 2000 125 421 426 11117967 10.1017/s0950268899004392 PMC2869616

[B19] Gulland FW Doxey DL Scott GR Changing morphology of Eperythrozoon ovis Res Vet Sci 1987 43 88 91 3628991

[B20] Liu XF Tian H Hou SL Sun ZX Zang RX Li QJ Zhao WP Yang WH Survey of eperythrozoon infection in human and animals and the route of transmission Zhongguo Shou Yi Za Zhi 1997 23 23 24 in Chinese

[B21] Tai XZ Yang DX Qin LJ Yang HP Jin LP Qi GL Yun X Investigation and analysis of eperythrozoon infection in human in Inner Mongolia Zhongguo Ren Shou Gong Huan Bing Za Zhi 1998 14 90 91 in Chinese

[B22] Huang ZM Ma XW Huang JM Zhao W Gong JY Wang KW An epidemiological investigation of eperythrozoon infection in human in Yuxi, Yunnan Zhongguo Gong Gong Wei Sheng 1999 15 31 in Chinese

[B23] Dong CZ Yan WY Liu CC Wei XJ Huang RL An epidemiological investigation of eperythrozoon infection in human in Sha County Zhonghua Yu Fang Yi Xue Za Zhi 2000 34 158 in Chinese

[B24] Yang XC Zhang ZC Sui Y An epidemiological investigation of eperythrozoon infection in human in Xuzhou Yu Fang Yi Xue Wen Xian Xin Xi 2000 6 39 in Chinese

[B25] Li ZY Wang ZH Yang YZ Guo L Li GL Liu XQ Survey of eperythrozoon infection in human and animals in Fuyang Zhongguo Gong Gong Wei Sheng 2001 17 46 in Chinese

[B26] Zhao LY Bai JW Characteristics of eperythrozoon in human under the electron microscope and clinical manifestation Neimenggu Yi Xue Za Zhi 2001 33 108 111 in Chinese

[B27] Tao XR Wang XJ Sun T Cui S Zheng DM Li Z Feng KJ Hu B Su JY An epidemic investigation of eperythrozoonsis in Shandong province Zhonghua Liu Xing Bing Xue Za Zhi 2001 22 359 361 in Chinese 11769692

[B28] Zhang MJ Ling XJ Feng YM Analysis of eperythrozoon infection in residents and outpatients in Yinchuan Ningxia Yi Xue Yuan Xue Bao 2002 24 60 61 in Chinese

[B29] Chen FY Zhang F Yao W Yuan Y Yang SH Wang BR Hu B Wnag YH Cui Y Zheng LL Li GY Jin FJ Survey of eperythrozoon infection in human in Dalian Zhongguo Ren Shou Gong Huan Bing Za Zhi 2003 19 100 132 in Chinese

[B30] Zhou XC He GR Zou DR Huang XZ Wang DJ Wen ZY Wang CQ Wu M Investigation of eperythrozoon infection in the Xingshan part of the Three Goarges Reservoir areas Zhongguo Ji Sheng Chong Bing Fang Zhi Za Zhi 2003 16 I9 in Chinese

[B31] Shi QG Li SZ Yang G Chen HZ Lu YZ Qi BB Hong Y The infection and prevention of eperythrozoon in altiplano Xizang Yi Yao Za Zhi 2007 28 3 6 in Chinese

[B32] Zhou XC Wang CQ Wang DJ Pan HM Zou XL Huang XZ Wu KQ Xu ZS He GR Zheng L An epidemiological investigation of eperythrozoonsis in Xingshan county of the Three Goarges Reservoir areas Zhongguo Mei Jie Sheng Wu Xue Ji Kong Zhi Za Zhi 2007 18 234 237 in Chinese

[B33] He CT Feng YS Li YL Zhao J Chen YX An epidemiological investigation of eperythrozoon infection in human in Gongshan, Yunnan Shi Yong Yu Fang Yi Xue 2007 14 382 383 in Chinese

[B34] Li FX Wang HR Deng XC An epidemiological investigation of eperythrozoon infection in human in Wendeng Zhongguo Cheng Xiang Qi Ye Wei Sheng 2008 2 58 in Chinese

[B35] Zhu M Cai L Wang LY Wang ZY Shen L Cai FZ Fei SJ Factors analysis of human Eperythtozoon infection in Shanghai Zhongguo Bing Yuan Sheng Wu Xue Za Zhi 2008 3 499 501 in Chinese

[B36] Han ZQ Zhao XH Yu AL Li D Li XM An epidemiological investigation of eperythrozoon infection in human in Taian Zhongguo Ren Shou Gong Huan Bing Xue Bao 2009 25 93 94 in Chinese

[B37] Qiu ZW Liu YK Qiu XQ Su MJ Zhao JY An analysis of eperythrozoon detection results in 18 316 petrochemical workers and staff members in Maoming Baotou Yi Xue Yuan Xue Bao 2010 26 16 17 in Chinese

[B38] Huang YX Preliminary investigation of eperythrozoon infection in human and animals Xu Qin Ye 2010 10 58 59 in Chinese

[B39] Deng J Yang XH Wang H A survey on eperythrozoon infection in the population from Hangzhou city, Zhejiang province Zhonghua Liu Xing Bing Xue Za Zhi 2010 31 647 649 in Chinese 21163095

[B40] Zhang Y Zhang ZL Yin JY Lv J Dong XC Shen TF Li D An investigation of eperythrozoon infection in a village, Liaoning province Zhonghua Liu Xing Bing Xue Za Zhi 2010 31 831 833 in Chinese 22993795

[B41] Wang Y Wang ZY Liu JZ Yan ZG Li YJ Zhang D Qin XL Yang DB Niu XD Ma J Wang YT Study on the spread approach in eperythrozoonosis of dairy cow Zhonghua Liu Xing Bing Xue Za Zhi 2007 28 311 312 in Chinese 17649674

[B42] Yuan CL Liang AB Yao CB Yang ZB Zhu JG Cui L Yu F Zhu NY Yang XW Hua XG Prevalence of Mycoplasma suis (Eperythrozoon suis) infection in swine and swine-farm workers in Shanghai, China Am J Vet Res 2009 70 890 894 19566474 10.2460/ajvr.70.7.890

[B43] Hoelzle LE Felder KM Hoelzle K Porcine eperythrozoonosis: from Eperythrozoon suis to Mycoplasma suis Tierarztl Prax Ausg G Grosstiere Nutztiere 2011 39 215 220 in German 22138829

[B44] Hoelzle LE Helbling M Hoelzle K Ritzmann M Heinritzi K Wittenbrink MM First LightCycler real-time PCR assay for the quantitative detection of Mycoplasma suis in clinical samples J Microbiol Methods 2007 70 346 354 17586075 10.1016/j.mimet.2007.05.009

[B45] Tasker S Peters IR Mumford AD Day MJ Gruffydd-Jones TJ Day S Pretorius AM Birtles RJ Helps CR Neimark H Investigation of human haemotropic Mycoplasma infections using a novel generic haemoplasma qPCR assay on blood samples and blood smears J Med Microbiol 2010 59 1285 1292 20651038 10.1099/jmm.0.021691-0 PMC3090618

[B46] Barker EN Darby AC Helps CR Peters IR Heesom KJ Arthur CJ Crossett B Hughes MA Radford AD Tasker S Molecular characterization of the uncultivatable hemotropic bacterium Mycoplasma haemofelis Vet Res 2011 42 83 21749699 10.1186/1297-9716-42-83 PMC3146833

